# Does training family physicians in shared decision making promote optimal use of antibiotics for acute respiratory infections? Study protocol of a pilot clustered randomised controlled trial

**DOI:** 10.1186/1471-2296-8-65

**Published:** 2007-11-29

**Authors:** France Légaré, Michel Labrecque, Annie LeBlanc, Robert Thivierge, Gaston Godin, Claudine Laurier, Luc Côté, Annette M O'Connor, Nadine Allain-Boulé, Jean Rousseau, Sylvie Tapp

**Affiliations:** 1 Research Centre of the Centre Hospitalier Universitaire de Québec, Québec, Canada; 2 Department of Family Medicine, Université Laval, Québec, Canada; 3 Faculty of Medicine, Université de Montréal, Montréal, Québec, Canada; 4 Faculty of Nursing, Pavillon Paul-Comtois, Université Laval, Québec, Canada; 5 GRIS/Faculty of Pharmacy, Université de Montréal, Montréal, Québec, Canada; 6 Faculty of Health Sciences, School of Nursing, University of Ottawa, Ottawa, Canada

## Abstract

**Background:**

In North America, although it varies according to the specific type of acute respiratory infections (ARI), use of antibiotics is estimated to be well above the expected prevalence of bacterial infections. The objective of this pilot clustered randomized controlled trial (RCT) is to assess the feasibility of a larger clustered RCT aiming at evaluating the impact of DECISION+, a continuing professional development (CPD) program in shared decision making, on the optimal use of antibiotics in the context of ARI.

**Methods/design:**

This pilot study is a cluster RCT conducted with family physicians from Family Medicine Groups (FMG) in the Quebec City area, Canada. Participating FMG are randomised to an immediate DECISION+ group, a CPD program in shared decision making, (experimental group), or a delayed DECISION+ group (control group). Data collection involves recruiting five patients consulting for ARI per physician from both study groups before (Phase 1) and after (Phase 2) exposure of the experimental group to the DECISION+ program, and after exposure of the control group to the DECISION+ program (Phase 3). The primary outcome measures to assess the feasibility of a larger RCT include: 1) proportion of contacted FMG that agree to participate; 2) proportion of recruited physicians who participate in the DECISION+ program; 3) level of satisfaction of physicians regarding DECISION+; and 4) proportion of missing data in each data collection phase. Levels of agreement of the patient-physician dyad on the Decisional Conflict Scale and physicians' prescription profile for ARI are performed as secondary outcome measures.

**Discussion:**

This study protocol is informative for researchers and clinicians interested in designing and/or conducting clustered RCT with FMG regarding training of physicians in shared decision making.

**Trial Registration:**

ClinicalTrials.gov Identifier: NCT00354315

## Background

Most health decisions occur in context of scientific uncertainty, the "grey zone" of decision making, and making decisions about the use of antibiotics for acute respiratory infections (ARI) is no exception [1]. ARI are the most frequently reported reasons for consulting a primary care provider in North America and include acute otitis media, acute rhinosinusitis, acute pharyngitis, and acute bronchitis. Together, these infections, excluding pharyngitis but including chronic sinusitis and eustachian tube disorders account for 5.7% of the primary diagnosis groups for adults and reach a total of 16.3% for children under the age of twelve in community based practices [2]. Although each type of ARI has distinct features, they all share in common that a large proportion of those infections are viral. Indeed, only 38% of adults with rhinosinusitis, 6% to 18% of children with ARI, and 5 to 15% of adults with pharyngitis have a bacterial infection [3,4]. Nonetheless in North America, although it varies according to the specific type of ARI [5-13], the use of antibiotic is estimated to be 63% to 67%, well above the expected prevalence of bacterial infections [5,7-11,14-17].

As suggested above, diagnostic and management of ARI are ripped with uncertainty. The probabilistic nature of the diagnosis of ARI makes it difficult for both the physician and the patient to choose the "best" course of action and is associated with prescription of antibiotics [18]. Scientific evidence that imparts conflicting results regarding treatment options (i.e. balance between risks and benefits) or the absence or insufficiency of scientific evidence makes this choice even more difficult [19]. Moreover, the probabilistic aspect of the evidence that is drawn from populations implies uncertain outcomes for the individual [20]. Consequently, patients and physicians need help in addressing their decisional needs and in resolving uncertainty when making decisions about the use of antibiotics for ARI, the most frequent "grey zone" health decisions in primary care.

Shared decision making (SDM) is a promising strategy for effective knowledge translation (KT) between health providers and their patients, particularly so in contexts of "grey-zone" decisions [21]. SDM refers to a process by which a healthcare choice is made by practitioners together with the patient, based on the best available evidence and in line with what an informed patient would value. If indeed "Knowledge translation is the exchange, synthesis and ethically-sound application of knowledge – within a complex system of interactions", then conceptualizing and operationalizing it as an interactive and dynamic process between patients and health providers has important consequences. It may foster a new breed of more effective KT interventions in clinical contexts [22].

However, it is not clear how SDM can be implemented in clinical practice. Given that most current efforts in KT deal with the implementation of clinical practice guidelines (CPG) where recommendations are considered as rules to be followed, it is not clear if the implementation processes of SDM and CPG are compatible. The proposed study addresses the urgent need to reconcile the current KT endeavours with the need to determine effective ways by which patients are engaged to share their preferences and participate in the process to achieve "grey zone" decisions. In other words, it lays the grounds for a new KT model at the clinical level: the SDM model.

**Figure 1:**
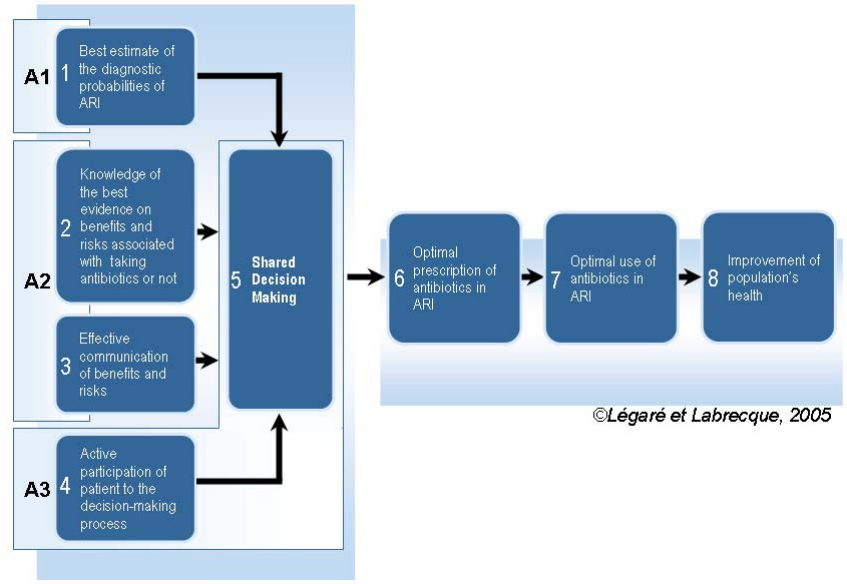
Decision+ Conceptual Framework.

We are planning a large clustered randomized clinical trial (RCT) aiming at evaluating the impact of DECISION+, a continuing professional development program in shared decision making on the optimal use of antibiotics for treating ARI. We report on the study protocol of an ongoing pilot clustered randomized controlled trial (RCT) to assess the feasibility of this larger trial. Based on our conceptual framework (Figure 1), specific aims include to: 1) assess the feasibility of recruiting Family Medicine Groups (FMG), family physicians/general practitioners (FPs/GPs) and their patients, 2) develop, adapt and validate DECISION+ training workshops and related material, and 3) evaluate physicians' participation and satisfaction regarding DECISION+. Specific aims also include estimating the impact of DECISION+ on: 1) decision of both physicians and their patients regarding the use of antibiotics, 2) decisional conflict scores of both physicians and their patients as well as level of agreement between their decisional conflict scores, 3) prescription profile of antibiotics for ARI, 4) intention of physicians to implement SDM in their clinical practice, 5) intention of physicians to implement clinical practice guidelines on ARI, 6) scores obtained by physicians on a Script Concordance Test (SCT), and 7) decisional regret of patients.

## Methods/Design

### Design

**Figure 2:**
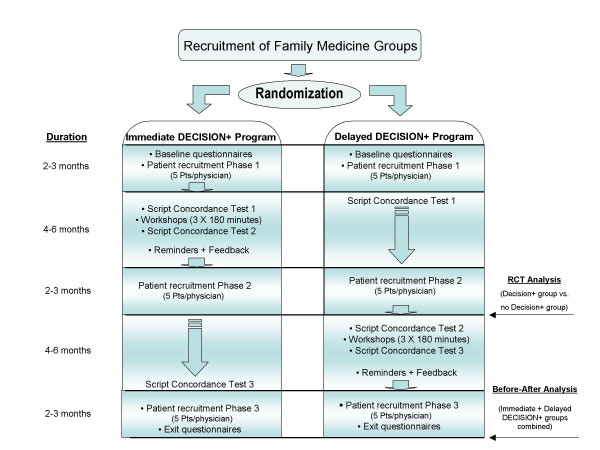
Decision+ Trial Design.

The study is a two-arm clustered RCT conducted in the Quebec City area, Canada (Figure 2). FMG is the unit of randomisation. A FMG consists in a group of family physicians who work in close cooperation with nurses to offer family medicine services to registered individuals. There are currently over 130 such groups in the Province of Quebec. FMG are randomized to either 1) an immediate DECISION+ program (experimental group) or 2) a delayed DECISION+ program (control group). Simple randomisation and concealed assignment is performed by an independent biostatistician using Internet-based software.

### Participants

#### Physicians

FPs/GPs are the main target participants and are recruited through FMG. A random list of all FMG in the Quebec City area was generated. Based on that list and in sequence, each physician who is in charge of the FMG is contacted by phone by one of the two co-principal investigators (FL or ML). If the contacted FMG includes at least 10 physicians, the study protocol is explained to the physician in charge and willingness to organise a meeting with colleagues from his/her group to further explain the nature of the project is sought.

FPs/GPs are not eligible to participate if they are currently participating or have previously participated to an implementation study of SDM in clinical practice. They are also excluded if they do not plan to be in clinical practice for the whole duration of the study (e.g. pregnancy, retirement, practice restricted exclusively to administrative duties). A FMG is not eligible if less than six eligible physicians agree to participate in the study. Reasons for exclusion or refusal to participate after the first telephone contact or after the face-to-face meeting are documented.

#### Patients

Patients are included if they meet the following criteria: a) adults or children consulting a participating physician for ARI, b) antibiotic treatment considered by the patient himself (or guardian) or by the physician, c) able (adults or guardians) to read, understand, and write French (equivalent to grade 8), and d) able to give informed consent (adults or guardians). Patients with a condition requiring emergency care are excluded. All participants, FPs/GPs and patients, sign an informed consent form approved by the ethics committee from the research institution.

#### Interventions

DECISION+ is a multifaceted CDP program offered over a 4- to 6-month period and includes the following:

#### Interactive workshops

Three 180-minute workshops are offered to participating FPs/GPs. Each one addresses a key component of our conceptual framework (Figure 1). The first workshop focuses on the probabilistic nature of the diagnosis of a bacterial vs. viral ARI. The second workshop focuses on the available evidence regarding the balance between risks and benefits when facing decision about the use antibiotics or not in ARI. It also addresses effective strategies to communicate this information to a patient. The third workshop focuses on strategies to foster active participation of patients in the decision-making process.

All three workshops include videos that facilitate group discussion about perceived barriers and facilitators to implementing SDM in the context of the use antibiotics for ARI. All workshops are offered at the FMG clinical site and leaded by experienced trainers (co-PI and co-I). Participants receive a toolkit including the material from the training workshops and a series of decision support tools to help them communicate with their patients the probability of a bacterial vs. viral ARI and the risks and benefits associated with the use of antibiotic in ARI. These decision support tools also include a section to help clarify patient's values regarding the use of antibiotics in ARI. All workshops are audio-taped to ensure fidelity of the intervention across FMG.

#### Reminders of expected behaviours

After completion of a given workshop, participating physicians receive reminders of the expected behaviours on a regular basis. The reminders summarize and emphasize the key elements of the workshops.

#### Feedback

Results of score agreement on the Decisional Conflict Scale between patients and physicians, as measured during the patient recruitment periods before the first workshop (Phase 1 in the experimental group and Phase 1 and 2 in the control group – see Figure 2), are provided to participating FPs/GPs in a personalized sealed envelope at the beginning of the third workshop. Physicians are also provided with pooled results from their colleagues. Time is provided during the workshop to explain how to interpret the results and for group discussion. A personalized and group prescription profile related to antibiotic prescribed to patients consulting with ARI is provided at the end of the series of workshops.

### Primary outcome measures

Primary outcome measures intend to assess the feasibility of a larger RCT aiming at evaluating the impact of the DECISION+ program. They include: a) proportion of contacted FMG that agree to participate, 2) proportion of recruited FPs/GPs that participate in the DECISION+ program, 3) level of satisfaction of FPs/GPs regarding DECISION+, and 4) proportion of missing data in each data collection phase. The subsequent RCT will be considered feasible if: 1) the proportion of contacted FMG that participate to the pilot study is 50% or greater, 2) the proportion of recruited physicians who participate in all three workshops is 70% or greater, 3) the mean level of satisfaction regarding the workshops is 65% or greater, and 4) the proportion of missing data in each completed questionnaire is less than 10%.

### Secondary outcome measures

Secondary outcomes measures aim at exploring the impact of the DECISION+ program on the decision making processes and the optimal use of antibiotics in the context of ARI. These measures include:

#### Decision to use antibiotics

After each clinical encounter for ARI, both patients and their FPs/GPs indicate if it was decided to: 1) use antibiotics, 2) delay antibiotics or 3) not use antibiotics.

#### Decisional conflict

After each clinical encounter for ARI, both patients and physicians complete the Decision Conflict Scale. This scale includes 16 items, each item being scored on a 5-point Likert scale (1 = strongly agree to 5 = strongly disagree) with higher score associated with higher decisional conflict. Internal consistency coefficients (Cronbach's alpha) from previous studies ranged from 0.78 to 0.92 in the patients' version and from 0.78 to 0.90 in the physicians' version [23,24].

#### Decisional regret

One to two weeks after clinical encounter, patients complete the Decisional Regret Scale over the phone (Cronbach's alpha = 0.81 to 0.92 in previous studies) [25].

#### Prescription profile of antibiotics in ARI

Prescription profile of all FPs/GPs from the Quebec City area, including all physicians participating in the study, is established using the Régie de l'Assurance Maladie du Québec (RAMQ) databases. Data are restricted to patients covered by the public prescription drug insurance of the Province of Quebec (about half of the total population in the province). Type of antibiotics prescribed and date of prescription filling are matched with the reasons for encounter (for an ARI) as stated by physicians for billing purpose. Data collection extends from 3 months before the beginning of the study to 3 months after the end of the study.

#### Script Concordance Test (SCT)

Clinical reasoning is assessed with a SCT on diagnosis and management of ARI [26-31]. The same SCT is completed three times over the study period (Figure 2). In both groups, SCT are completed at the beginning of the first and at the end of the third workshop (SCT 1 and 2 in the experimental group and SCT 2 and 3 in the control group). SCT 1 in the control group is completed at baseline and SCT 3 in the experimental group at exit of the study.

#### Physicians' reactions to uncertainty

Two subscales from the Physicians' Reactions to Uncertainty questionnaire are completed at baseline: anxiety about uncertainty and disclosing uncertainty to patients [32,33].

#### Intention to engage in SDM and to use clinical practice guidelines regarding the use of antibiotics in ARI in future clinical encounters

At baseline, in reference to the theory of planned behaviour [34], FPs/GPs complete a questionnaire assessing their attitude, subjective norm, perceived behavioural control, and intention to engage in SDM as well as to use clinical practice guidelines in future clinical encounters dealing with the use of antibiotics in ARI. Physicians are also asked to complete this questionnaire at the exit of the study.

Before their clinical encounter for ARI, each participating patient is asked to complete a similar questionnaire on intention to engage in SDM in a clinical encounter dealing with the use of antibiotics in ARI.

### Data collection process

Data on FPs/GPs' socio-demographic characteristics, intention to integrate SDM and clinical practice guidelines on ARI in practice, and reaction to uncertainty are collected at baseline in both groups (Figure 2). Then, five patients consulting a participating physician for an ARI are recruited by a research assistant in the waiting room before the clinical encounter (Phase 1). Data on patients' socio-demographic characteristics and intention to engage in SDM when consulting for an ARI are collected at that point in time. FPs/GPs and patients are both invited to complete the Decisional Conflict Scale after the clinical encounter. Patients and FPs/GPs are blinded to the answers of the other member of the dyad. Patient recruitment and completion of the Decisional Conflict Scale by patient and physician are repeated at two weeks after the third workshop of the immediate DECISION+ group (Phase 2) and again after the third workshop of the delayed DECISION+ group (Phase 3). All patients are contacted by phone one to two weeks after their clinical encounter to complete the Decisional Regret Scale. Finally, all participating FPs/GPs complete an exit questionnaire including data on intention to engage in SDM and use of clinical practice guideline as well as on satisfaction with the DECISION+ program.

### Sample size and analysis

The objective is to recruit four FMG (at least 24 physicians and a total of 360 patients with an ARI that is 5 patients per physician in each one of the three recruitment phases) for this pilot clustered RCT. This study is a feasibility study and therefore no sample size calculation was performed. Our design allow a comparison of physicians (and patients) data randomly exposed or not to the DECISION+ program (RCT) and a comparison of before and after data combining the results of the immediate and delayed exposure to the DECISION+ program.

It is not possible to blind participating FPs/GPs from the planned intervention but individuals involved with data analyses will be blinded to group allocation and initial results will be provided to researchers without revealing group allocation. Statistical analysis for the primary outcome feasibility measures will be performed using proportions and means and associated 95% confidence intervals. Methods for assessing the statistical significance of the secondary outcome measures between study groups will take into account the clustered nature of the dataset.

## Discussion

This study protocol is informative for researchers and clinicians interested in designing and/or conducting clustered RCT regarding training of family physicians in shared decision making. In line with our previous work [35,36], this pilot clustered RCT is highly relevant to family medicine as ARI are the most frequently reported reasons for consulting a primary care provider in North America and the use of antibiotics for ARI appears to be suboptimal. Although this is a pilot project, the deliverables are many and important as they will: 1) provide innovative insight on how to successfully implement change in clinical practices using a SDM perspective, 2) be helpful for future research on new models of collaborative care within the workforce environment in primary care, 3) serve as a strategy to increase quality of care and patient safety, and 4) reinforce a patient-centered care approach, one that highly values relationships and a shared understanding by patients and their FPs/GPs of the nature of the problem that is being addressed, the available options, and the role of both patient and provider to the decision making process [37].

In summary, this is the first study to assess the impact of DECISION+, a multifaceted CDP program that fosters SDM during primary care clinical encounters dealing with the decision to use antibiotics or not in ARI. Its results will lay the grounds for the implementation of SDM in family medicine. The expected results from this project should guarantee the feasibility of a larger clustered RCT aiming at evaluating the impact of DECISION+ on the optimal use of antibiotics use in ARI.

## Competing interests

The authors declare that they have no competing interests.

## Authors' contributions

FL and ML conceived the study and validated the methods. ML, FL, LC, GG, CL, AOC and RT sought funding and ethical approval. FL, ML and JR are responsible for the management of the trial. FL, ML, JR, AL and NAB collectively drafted the current manuscript. All authors contributed to the research design, read, and approved the final manuscript. FL and ML are its guarantors.

## Pre-publication history

The pre-publication history for this paper can be accessed here:


